# Autonomic Nervous System Activity before Atrial Fibrillation Onset as Assessed by Heart Rate Variability

**DOI:** 10.31083/RCM25364

**Published:** 2025-01-08

**Authors:** Jean-Marie Grégoire, Cédric Gilon, François Marelli, Pascal Godart, Hugues Bersini, Stéphane Carlier

**Affiliations:** ^1^IRIDIA, Université Libre de Bruxelles, 1050 Bruxelles, Belgium; ^2^Cardiology Department, Université de Mons, 7000 Mons, Belgium; ^3^ISIA Lab, Université de Mons, 7000 Mons, Belgium; ^4^CHU Helora, Site Kennedy, 7000 Mons, Belgium

**Keywords:** atrial fibrillation, heart rate variability, autonomic nervous system, spectral analysis neuromodulation

## Abstract

**Background:**

Neuromodulation has been shown to increase the efficacy of atrial fibrillation (AF) ablation procedures. However, despite its ability to influence the autonomic nervous system (ANS), the exact mechanism of action remains unclear. The activity of the ANS via the intracardiac nervous system (ICNS) can be inferred from heart rate variability (HRV). Therefore, this study aims to investigate the significance of changes in the ICNS prior to the onset of AF by analyzing the evolution of HRV in a large new cohort of patients.

**Methods:**

We selected and annotated recordings with AF and atrial flutter from our database of 95,871 Holter recordings. Each recording included both sinus rhythm and one or more AF episodes. We computed parameters estimating parasympathetic activity (root mean square of successive RR interval differences (RMSSD) and percentage of successive RR intervals that differ by more than 50 ms (pNN50)), as well as HRV frequential parameters a few minutes before AF onset. To allow a minute-by-minute assessment of the parameter changes, we computed their values over 5-minute sliding windows, starting at 35 minutes before AF onset.

**Results:**

The mean age of the whole group of patients was 71.1 ± 11.3 years (range 35–99), the total number of episodes was 1319 on 623 recordings from 570 patients, with an average of 2.1 ± 2.2 episodes per recording (range 1–17) and 2.3 ± 2.6 episodes per patient (range 1–21). The proportion of premature atrial contractions (PACs) increased from 4.8 ± 0.3%, 35 minutes before the onset of AF to 8.3 ± 0.4%, 5 minutes before the AF episode. We measured a statistically significant increase in very-low-frequency (VLF), low-frequency (LF), high-frequency (HF), RMSSD and pNN50 between 35 minutes and 5 minutes before AF onset.

**Conclusions:**

Our data suggest that a significant short-term increase in vagal activity precedes most AF events. Dynamic changes in HRV parameters could be considered when determining the optimal neuromodulation strategies.

## 1. Introduction

Neuromodulation techniques are increasingly used in atrial fibrillation (AF) ablation. However, they have yielded conflicting and sometimes disappointing results in clinical trials, which might be related to the lack of site-specific targeting and appreciation of complex neural circuitry. Their mechanism of action remains to be fully elucidated, as recent studies seem to yield paradoxical results: for example, stimulation of vagal activity is used to reduce AF crises [1], but it was also shown that the addition of ganglionated plexi ablation (GPA) to pulmonary vein isolation (PVI) reduces the AF recurrence rate despite the elimination of a large proportion of parasympathetic cell structures [2].

The action of the autonomic nervous system (ANS) can be estimated from heart rate variability (HRV), although the complexity of the relationship between HRV and the ANS complicates the analysis [3]. HRV assessment may involve spectral analysis, statistical methods, nonlinear systems studies, machine learning techniques, deceleration capacity, turbulence and fragmentation indices, reflecting the influence of multiple regulatory mechanisms operating on different time scales.

The objective of this retrospective clinical study was to investigate the significance of changes in the intracardiac nervous system (ICNS) before the onset of AF by following the evolution of HRV parameters in a large new cohort of patients.

## 2. Materials and Methods

All the recordings with AF and atrial flutter that we annotated to serve as ground truth for this study were selected from our database of 95,871 Holter recordings. The inclusion criteria for this study were as follows: adults aged over 35 years with at least one AF event detected by Holter. The exclusion criteria were the presence of a cardiac implanted electronic device (CIED), and persistent/permanent AF. This database contains all available Holter recordings from December 2009 to December 2019 for outpatients and inpatients from 4 centers (3 hospitals and one private clinic). The Holter recording system used consisted of two-channel Spiderview digital recorders (Microport CRM, Bagneux, France). Recordings were transferred from the recorders to the Microport analysis software Synescope (version 3.30a, Microport CRM, Bagneux, France) for an initial correction to eliminate the coarsest artifacts of the complexes. All recordings were subsequently edited and visually reanalyzed in their entirety to search for all AF and atrial flutter episodes longer than 30 seconds. Recordings with atrial tachycardias were excluded. A total of 1319 paroxysmal AF episodes from 570 patients were found and labeled, with each recording including both sinus rhythm and one or more AF episodes.

For the computation of HRV parameters, we studied only sustained AF episodes with a duration of at least 5 minutes and preceded by at least 35 minutes of normal sinus rhythm (NSR), which represents a total of 880 episodes. We used the gradient-based detection method implemented in the NeuroKit2 Python toolbox [4] to automatically label the R-peaks in the selected electrocardiograms (ECG). We then filtered the obtained RR intervals using a local variability threshold to detect ectopic beats, misdetections and other artefacts [5]. We removed these detected outliers and the immediately following RR intervals by linearly interpolating their values, to prevent them from corrupting the HRV parameters.

We calculated two temporal parameters to estimate parasympathetic activity a few minutes before AF onset: root mean square of successive RR interval differences (RMSSD) and percentage of successive RR intervals that differ by more than 50 ms (pNN50) [6]. We also computed HRV frequential parameters to complete our analysis, using the Fourier transform of the time series of heartbeats to obtain the power spectrum of the ECG signal. The following spectral variables were calculated in both absolute (ms^2^) and normalized units (NU): very-low-frequency (VLF) components (<0.04 Hz); low-frequency (LF) components (0.04–0.15 Hz); high-frequency (HF) components (0.15–0.40 Hz). We then calculated the LF/HF ratio, which is often used heuristically as an index of the modulation of sympatho-vagal interactions [7]. We obtained power in normalized units by dividing the values by the total power of the spectrum minus the VLF components [8]:

Power ⁢[NU]=100× Power/(Total Power - VLF)

We used the Neurokit2 Python toolbox to compute all listed HRV parameters.

**Fig. 1.**
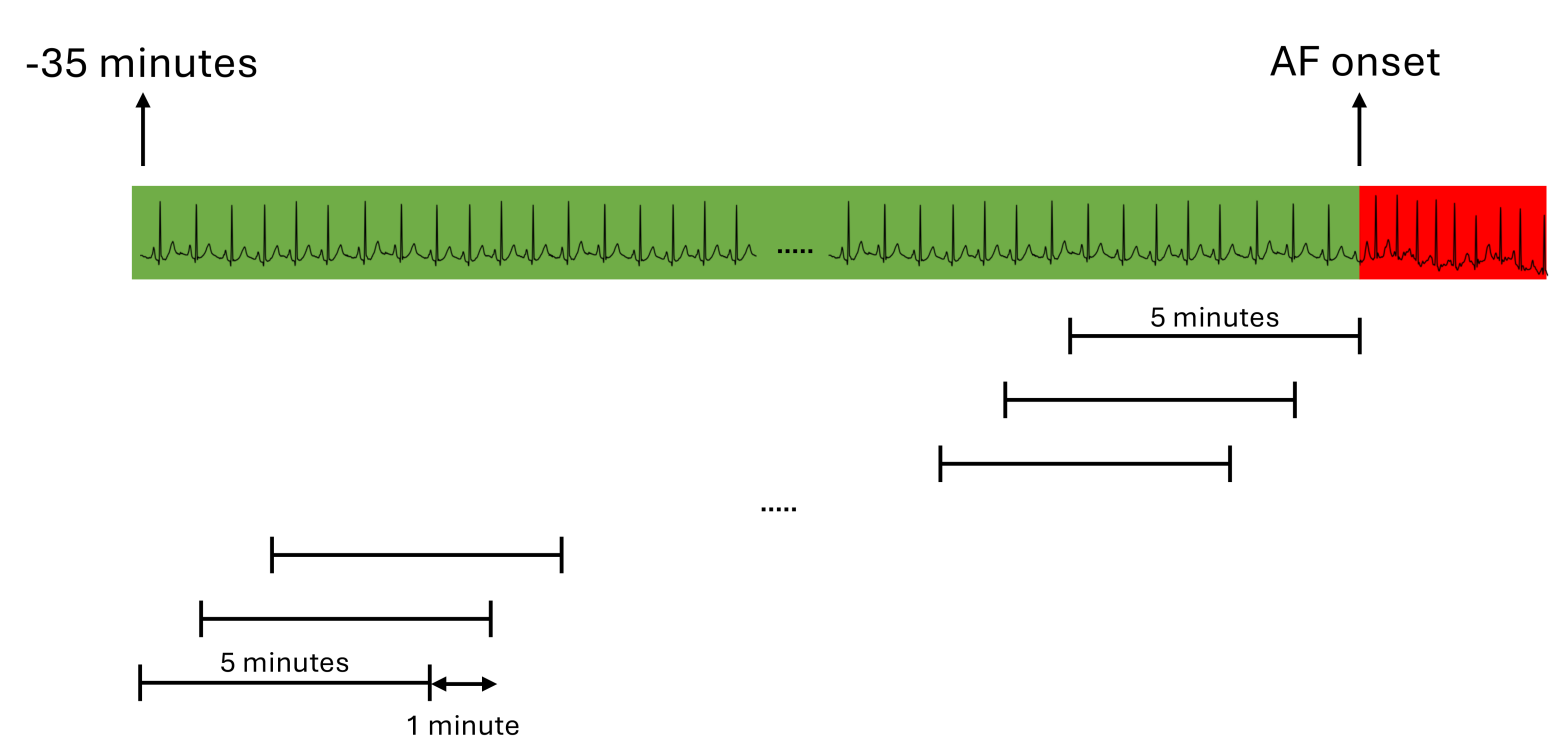
**Sliding computation windows to capture the changes in heart rate variability (HRV) parameters**. Values over a 5-minute window are computed, then the computation window is shifted 1 minute further and the process is repeated. The evolution of HRV parameters is thus plotted over the 35 minutes preceding atrial fibrillation (AF) onset with a 1-minute resolution. The electrocardiogram (ECG) strip shown is not at scale and only serves as visual help.

To observe the evolution of these parameters in the minutes preceding the AF onset, we used sliding windows for our computations. Starting 35 minutes before the onset, we calculated all the parameters over 5-minute windows, offsetting the start and end of the computation window by 1 minute each time we calculated a new value, as illustrated in Fig. 1. This allowed us to dynamically assess the changes in HRV parameters with a 1-minute resolution, starting with a window spanning from 35 to 30 minutes before the AF onset, and ending with a window covering the 5 minutes immediately preceding the onset. We chose to use 5 minutes for the computation windows as we have shown in previous work that it is the optimal length for predicting AF episodes [9]. 5-minute time frames are also commonly used to assess the significance of subclinical AF [10].

We carried out a statistical analysis of the HRV parameters to assess the significance of their evolution over time. For each parameter in each time window, we computed the mean and the standard error of the mean (SEM) over all AF episodes. We then computed *p*-values via the Mann-Whitney U test using the SciPy library in Python [11].

## 3. Results

The mean age of the whole group of patients was 71.1 ± 11.3 years (range 35–99), the total number of episodes was 1319 in 623 records from 570 patients, the number of episodes per record was 2.1 ± 2.2 (range 1–17) and the number of episodes per patient was 2.3 ± 2.6 (range 1–21). The proportion of premature atrial contractions (PACs) increased from 4.8 ± 0.3%, 35 minutes before AF onset to 8.3 ± 0.4%, 5 minutes before the AF crisis.

**Fig. 2.**
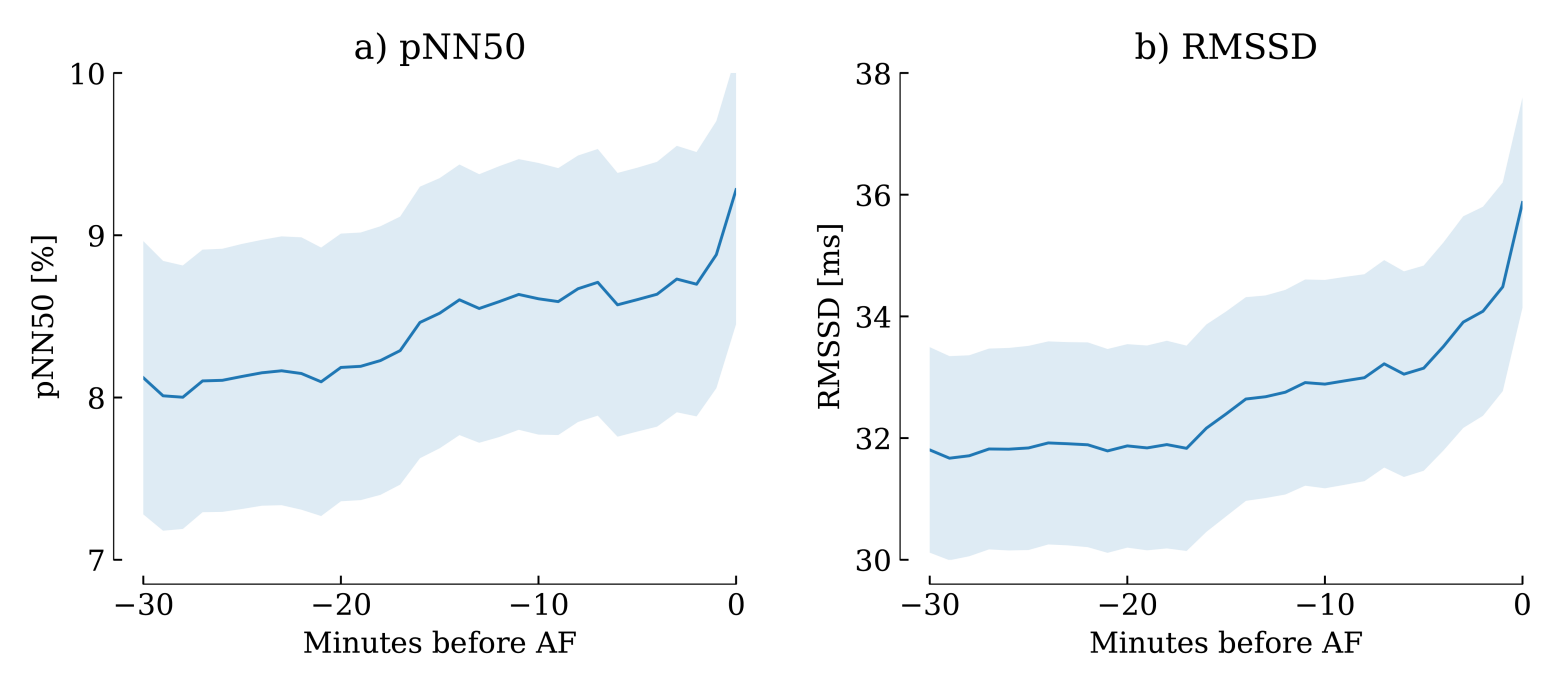
**The evolution of pNN50 and RMSSD over the minutes preceding AF onset was progressive but significant**. Shaded areas indicate 95% confidence intervals. (a) pNN50 evolution before AF onset. (b) RMSSD evolution before AF onset. pNN50, percentage of successive RR intervals that differ by more than 50 ms; RMSSD, root mean square of successive RR interval differences; AF, atrial fibrillation; pNN50, percentage of successive RR intervals that differ by more than 50 ms.

**Fig. 3.**
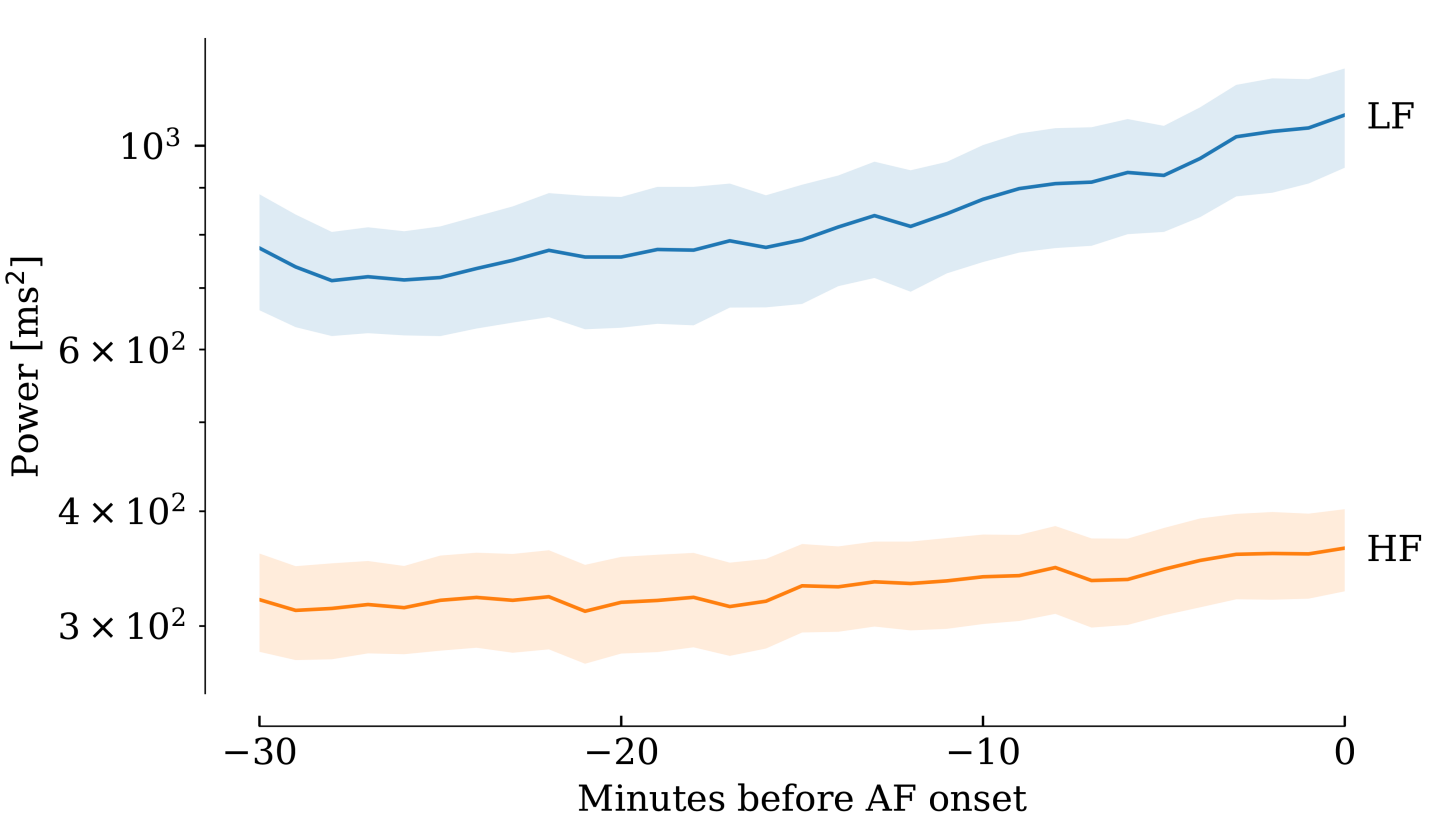
**The HF (orange) and LF (blue) components of the power spectrum both increased consistently during the minutes preceding AF onset**. Shaded areas indicate 95% confidence intervals. HF, high-frequency; LF, low-frequency; AF, atrial fibrillation.

**Fig. 4.**
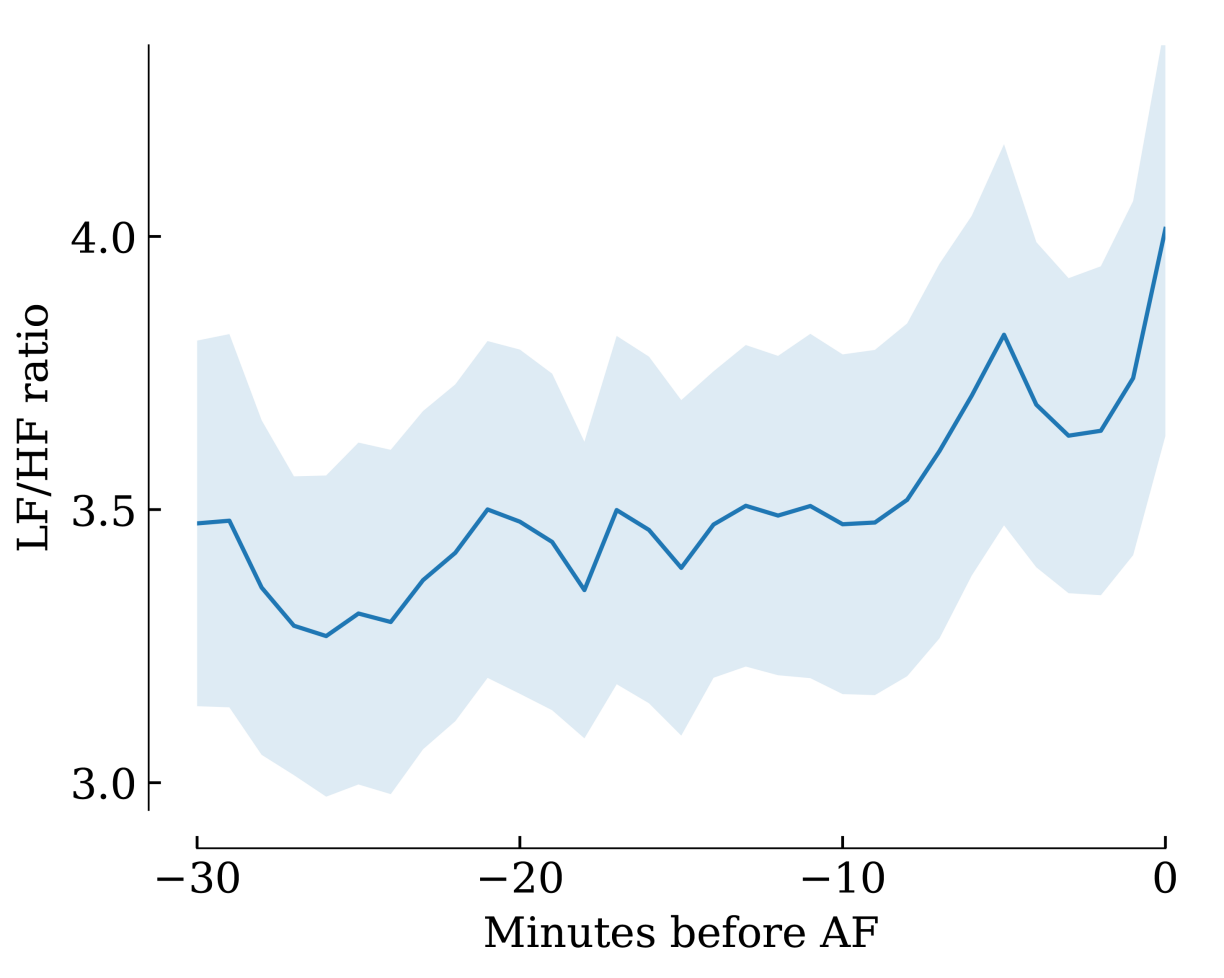
**The LF/HF ratio increased significantly over the minutes preceding AF onset**. Shaded areas indicate 95% confidence intervals. LF/HF, low-frequency/high-frequency; AF, atrial fibrillation.

Table 1 contains the computed temporal and spectral HRV parameters in the 5-minute windows located 35 minutes before and 5 minutes before the AF onset. With a threshold *p*-value < 0.05, there was a statistically significant increase in all parameters, although the increased mean heart rate was only borderline significant. Figs. 2,3,4, derived from the sliding computation windows, show that pNN50, RMSSD, LF, HF and LF/HF progressively increased over the 30 minutes preceding the AF event.

**Table 1.** **Changes in temporal and spectral values 35 and 5 minutes before the onset of AF**.

		From –35^′^ to –30^′^	From –5^′^ to AF onset	*p*-value
RR (ms)		926.55 ± 6.15	914.05 ± 6.63	0.0485
	pNN50 (%)	8.12 ± 0.42	9.27 ± 0.41	<0.0001
	RMSSD (ms)	31.81 ± 0.84	35.88 ± 0.86	<0.0001
	LF/HF	3.48 ± 0.17	4.01 ± 0.19	0.0007
LF (ms^2^)		770.07 ± 55.66	1072.52 ± 66.19	<0.0001
	LF (NU)	60.00 ± 0.74	63.56 ± 0.73	0.0004
HF (ms^2^)		319.83 ± 19.61	361.48 ± 18.44	0.0002
	HF (NU)	33.66 ± 0.61	30.72 ± 0.59	0.0005
VLF (ms^2^)		1619.31 ± 249.45	1771.43 ± 152.27	<0.0001

Changes in temporal and spectral HRV parameters were statistically significant (*p*-value < 0.05) between windows located 35 and 5 minutes before AF onset. AF, atrial fibrillation; pNN50, percentage of successive RR intervals that differ by more than 50 ms; RMSSD, root mean square of successive RR interval differences; LF, low-frequency; HF, high-frequency; VLF, very-low-frequency; RR, RR intervals; NU, normalized units.

A complete 24-hour assessment of HRV allows for the capture of circadian rhythm variations and long-term trends. Using the time periods of 8:00 AM to 8:00 PM for the daytime and 8:00 PM to 8:00 AM for the nighttime, we found that 45.23% of episodes occurred during the daytime and 54.77% occurred during the nighttime. This finding is similar to those of a recent study [12].

## 4. Discussion

This study reports an analysis of more than a thousand episodes of AF, making it the largest study of its kind to date. The key finding is that all HRV parameters measuring vagal activity significantly increase before the onset of AF, suggesting that a significant increase in parasympathetic activity precedes AF episodes in most patients.

Numerous studies have investigated the role of the ANS in triggering AF episodes. The conclusions of these studies are not always concordant, and it seems that patient cohorts are not comparable, particularly in terms of risk factors. Most studies reported Holter data from fewer than 100 patients. The authors either reported an increase in HF, LF, and the LF/HF ratio in all patients [8], divided patients into two groups (an increase in HF with a decrease in LF, or vice versa [13, 14]), or reported no significant variations in spectral values [15]. However, none of the studies focusing on temporal parameters have shown significant variations prior to the onset of AF episodes [16]. Some authors considered the type of underlying cardiac pathology, whereas other authors who analyzed the mode of presentation of extrasystoles achieved different results [17, 18, 19]. The disparity of published results emphasizes the difficulty of identifying general characteristics applicable to all patients. Indeed, the relatively small number of episodes included in those studies and the great heterogeneity of AF make it very difficult to draw global inferences about ANS behavior.

The ANS is an extremely complex structure [20]. An interconnected three-level hierarchy with numerous feedbacks regulates its interactions, culminating in and acting upon the ICNS, also known as the little brain of the heart [21]. The first level includes the cerebral cortex, brainstem, and spinal cord; the second level includes all intrathoracic but extracardiac ganglia; and the third level is the ICNS. The latter’s activity depends on several factors, including respiratory oscillations, pressure oscillations, metabolic processes, hormones, thermoregulation, and the angiotensin-converting enzyme (ACE) system. The ICNS itself is anatomically very complex and includes vagal cells and the axonal branches of sympathetic neurons. It integrates efferent sympathetic and parasympathetic activity, involving mechanosensitive and chemosensitive neurons that influence beat-to-beat changes. Due to this complexity, making inferences about the ANS from HRV parameters is a challenge. We must be aware of over-simplistic interpretations. However, one of the keys to differentiating between the actions of the parasympathetic and sympathetic systems lies in the speed of their action on heart rate: around one second for the parasympathetic system, and slower, in the region of around 5 seconds, for the sympathetic system [3, 22]. These two branches of the ANS constantly interact, one compensating for the activity of the other, thereby maintaining an autonomic balance [6].

It is challenging to gauge sympathetic activity solely on the basis of fluctuations in heart rate. Indeed, direct imaging of cardiac sympathetic innervation requires the use of radiolabeled sympathomimetic amines, such as 123-iodine-metaiodobenzylguanidine and 11-carbon-meta-hydroxyephedrine [23]. Direct measurement of adrenergic activity through variations in heart rate is not feasible, even though part of the LF peak frequency is due to impulses from the adrenergic system [3, 6, 22]. On the other hand, HRV seems to be a good measure of parasympathetic reactivity, since vagal activity is reflected in heart rate beat by beat.

We focused on variability parameters that reflect parasympathetic activity when computed over 5-minute periods. Temporal parameters related to vagal tone increase significantly in our patients over the 30 minutes preceding the onset of episodes. The frequential parameter HF, which reflects vagal tone, similarly increases over that same period. This frequency band is commonly called the respiratory band, as it corresponds to heart rate variations related to the respiratory cycle [24].

The LF band reflects a complex mix of sympathetic, vagal, and non-linear influences, showing the input of both the sympathetic and parasympathetic branches [3, 25]. Because of this, the significant increase in LFs in the 30 minutes preceding AF is very difficult to interpret on its own.

The VLF band also significantly increases over that period. However, since their precise nature is unclear, we cannot infer anything from it even if they appear to play some role. VLFs could correspond to slower influencing rhythms such as hormonal rhythms and thermoregulation [22]. It has been shown that, in dogs, VLFs occur with renin-angiotensin system blockade or LFs and HFs increase [26]. Experimental evidence suggests that the heart intrinsically generates the VLF rhythm, and that its amplitude and frequency are modulated by efferent activity of the central nervous system (CNS) due to physical activity and stress responses [27].

**Fig. 5.**
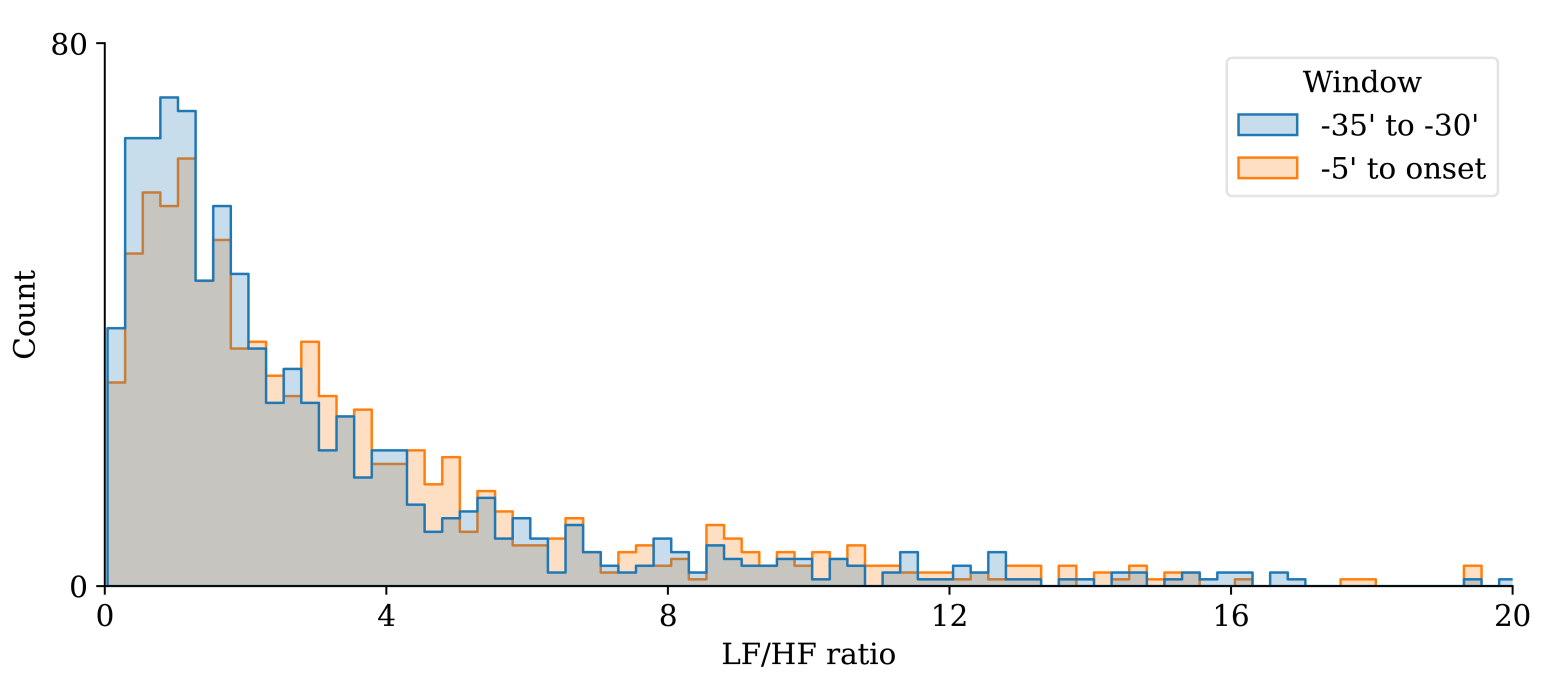
**LF/HF histograms demonstrating the absence of a clear delineation between the adrenergic and vagal systems**. This suggests that the concept of a distinct division between these two systems using the LF/HF ratio is not useful. LF/HF, low-frequency/high-frequency.

It has been suggested that the LF/HF ratio is a measure of ANS balance. However, there is no universally accepted “normal” or standard value for the LF/HF ratio due to variations in individual physiology and differences in measurement techniques, and this ratio should be interpreted with caution [28]. A few studies have attempted to use this ratio to differentiate vagal from adrenergic AF by thresholding [29, 30, 31]. Using their criteria, we found that the same patients often presented with both types of crises. Moreover, our analysis of the histogram of the LF/HF ratio in our patients revealed that it was not possible to correctly discriminate between these two types of AF. Indeed, as seen in Fig. 5, this histogram contained only one peak, regardless of the computation window being 30 minutes or immediately before the AF onset. We still measured an unexpected significant increase in the LF/HF ratio in the minutes preceding AF onset, which requires further investigation. It could be theorized that the adrenergic part of LF increases proportionally more than vagal activity. However, this remains a highly speculative proposition, given the difficulty of estimating sympathetic activity based solely on HRV.

The significant evolution of both temporal and frequential HRV parameters listed above seems to indicate that the dynamics of the ANS undergo substantial changes prior to the onset of AF. Such an increase in vagal activity has already been demonstrated in hypertensive patients who will experience AF [32]. Our data suggest that this phenomenon could be generalized to all at-risk patients.

Explaining the physiopathology behind these ANS modifications represents a significant challenge. For that purpose, one may employ Coumel’s Triangle, a conceptual model used to describe the underlying mechanisms of cardiac arrhythmias, and particularly AF [33]. This model delineates three indispensable elements that must interact for an arrhythmia to occur. The initial element is the substrate, atrial cardiomyopathy, which encompasses structural and functional abnormalities in heart tissue. An evaluation of this substrate may be conducted using the CHADVASC score [34]. The second element is the modulating factor, which is associated with the patient’s ANS status [35]. The third element is the trigger, which may be a rapid increase in vagal drive, potentially preceded by a stress factor [36].

We can only postulate a few possible hypotheses for the etiology of the observed augmentation in parasympathetic tone. It might result from an initial increase in sympathetic activity due to acute or chronic exposure to stressors [37, 38]. It might also be attributed to excessive physical exertion during sports for some patients [39]. Furthermore, we cannot exclude autonomic dysregulation as a cause [40, 41].

In our patients, the number of PACs increased progressively, reaching almost 9% just before the onset of AF episodes. In most cases, an AF crisis begins with a supraventricular extrasystole, and very rarely with a ventricular extrasystole. A useful framework for understanding arrhythmias is that they initially require a trigger to generate an extra stimulus. This extra stimulus needs to have the right properties and be perfectly timed within a vulnerable window to generate the ectopic beat ultimately responsible for AF onset. The increase in extrasystoles may be influenced by ANS modulations, but arrhythmia can only be triggered when a certain threshold of autonomic activity is reached. At that point, PACs have become abundant, and each additional premature contraction increases the likelihood of triggering an arrhythmia. The extrasystole (i.e., the extra stimulus) is an essential trigger, but it can only induce an arrhythmia if the ANS is in the right condition.

The counting and presentation of PACs have raised the hope of reducing the AF burden in CIED patients by means of anti-tachycardia algorithms [42, 43]. However, the lack of evidence of efficacy from all the clinical studies carried out to demonstrate the effectiveness of these algorithms has clearly shown that using only the presentation of PACs to treat AF does not work [44]. A probable explanation is that the increase in PACs before AF onset is consecutive to ANS modulations, which would imply that these algorithms act too late as the triggering process has already begun. Therefore, modulations of the ANS might be a more effective target to treat AF. Indeed, a recent study showed that overdrive pacing based on an indirect measure of ANS reactivity reduced the incidence of atrial high-rate episodes compared to conventional rate-adaptive pacing in patients with sinus node dysfunction [45].

As stated earlier, our observations support that ANS dynamics significantly evolve prior to AF onset, most notably through an overall increase in parasympathetic parameters. However, many questions remain unanswered. Although parasympathetic activity preceding AF onset increases on average, individual behavior may vary, and some patients may experience a predominant increase in sympathetic nerves instead. It is therefore important to guide therapy on a patient-by-patient basis. A purely pragmatic approach would be to analyze each patient’s Holter recordings to ascertain which branch of the ANS is most activated prior to the attacks. The most appropriate neuromodulation technique could then be applied to patients whose AF episodes are all the same type. Our results suggest, for example, that by analyzing the dynamics of the vagal activity for each individual patient using sliding windows, we could identify patients who might respond better to GPA associated with PVI.

### Limitations of this Study

This retrospective study involved patients undergoing a variety of treatments, some of which could influence HRV. Due to data anonymization, the specific treatments for most patients remained unknown. We focused on episodes exceeding 5 minutes to enhance the relevance of our findings for predicting AF episodes. Importantly, this analysis was based on real-world data, making it challenging to replicate the findings under controlled experimental conditions. To further validate these results, prospective studies are necessary, potentially involving tens of thousands of patients from at-risk groups, with prolonged ECG monitoring to account for the prevalence of AF episodes.

## 5. Conclusions

Short-term increased parasympathetic activity may be predictive of an impending AF event. Dynamic changes in HRV parameters could be considered to determine the best strategy for neuromodulation techniques.

## Availability of Data and Materials

The first part of the database can be downloaded from Zenodo: Cédric Gilon, Jean-Marie Grégoire, Marianne Mathieu, Stéphane Carlier, & Hugues Bersini (2023). IRIDIA-AF, a large paroxysmal atrial fibrillation long-term electrocardiogram monitoring database (1.0.1) [Data set]. Zenodo. https://doi.org/10.5281/zenodo.8405941.
